# Binge drinking: a pattern associated with a risk of problems of alcohol use among university students ^1^

**DOI:** 10.1590/1518-8345.1891.2925

**Published:** 2017-09-12

**Authors:** André Bedendo, André Luiz Monezi Andrade, Emérita Sátiro Opaleye, Ana Regina Noto

**Affiliations:** 2 Doctoral student, Departamento de Psicobiologia, Universidade Federal de São Paulo, São Paulo, SP, Brazil. Scholarship holder at Fundação de Amparo à Pesquisa do Estado de São Paulo (FAPESP), Brazil.; 3 PhD, Professor, Departamento de Psicologia, Universidade Anhembi-Morumbi, São Paulo, SP, Brazil.; 4 PhD, Researcher, Departamento de Psicobiologia, Universidade Federal de São Paulo, São Paulo, SP, Brazil.; 5 PhD, Adjunct Professor, Departamento de Psicobiologia, Universidade Federal de São Paulo, São Paulo, SP, Brazil. Scholarship holder at Conselho Nacional de Desenvolvimento Científico e Tecnológico (CNPq), Brazil.

**Keywords:** Alcohol Drinking, College Drinking, Binge Drinking, Internet, Students, Universities

## Abstract

**Objective:**

to evaluate problems associated with alcohol use among university students who reported binge drinking in comparison to students who consumed alcohol without binging.

**Method:**

a cross-sectional study among university students (N=2,408) who accessed the website about alcohol use. Logistic and linear regression models were included in the statistical analyzes.

**Results:**

alcohol use in the last three months was reported by 89.2% of university students; 51.6% reported binge drinking. Compared to students who did not binge drink, university students who presented this pattern were more likely to report all evaluated problems, among them: black out (aOR: 5.4); having academic problems (aOR: 3.4); acting impulsively and having regrets (aOR: 2.9); getting involved in fights (aOR: 2.6); drinking and driving (aOR: 2.6) and accepting a ride with someone who had drunk alcohol (aOR: 1.8). Students who binged also had higher scores on the Alcohol Use Disorders Identification Test (b=4.6; p<0.001), more negative consequences (b=1.0; p<0.001) and a reduced perception of the negativity of the consequences (b=-0.5; p<0.01).

**Conclusion:**

binge drinking was associated with an increase in the chances of manifesting problems related to alcohol use. The conclusions of this study cannot be generalized for all of the Brazilian population.

## Introduction

Brazil has more than 7.3 million university students^1^ who present a higher yearly and monthly prevalence of alcohol use^2^ ^-^ ^3^ than the general population. One of the patterns of alcohol use especially common among university/undergraduate students is binge drinking, which is defined as the intake of five or more doses/alcoholic beverages on one single occasion^3^. The use of alcohol has been associated with several risks and/or negative consequences, such as driving under the influence of alcohol, heart problems, violence, injuries (falls, poisoning, drowning, traffic accidents) and death^4^ ^-^ ^5^. It is estimated that 76% of the money spent in the United States on health in relation to excessive alcohol consumption is due to binge drinking^6^. In Brazil, one in four students reported binging during the past 30 days, indicating a particular group of students often exposed to the various risks associated to this drinking pattern^3^.

Assessing the consequences of alcohol consumption should not only consider the frequency of drinking, but also binging, which is a pattern associated with problems related to alcohol consumption^7^. In Brazil, a study carried out with university students found that binge drinking was associated with several negative consequences (drinking and driving, involvement in traffic accidents, missing university activities, low academic performance and involvement in fights or trouble with the law)^8^. However, that study only considered students from some courses in the health area of a single public higher education institution; and data indicate that the frequency of binge drinking varies according to the study area, the type of educational institution and the region of the country^3^. Thus, Brazilian studies comparing binge drinking and the consequences associated with alcohol consumption among samples of university students from courses of various study areas, educational institutions and regions of the country are necessary.

In this study, our objective was to evaluate the consequences and problems associated with alcohol consumption among university students who report binge drinking, in comparison to students who do not binge. The hypotheses of this study were: university students who consume alcohol by binging present: 1) higher scores in the AUDIT, 2) more negative consequences associated with alcohol use and 3) greater chances of reporting problems or consequences associated with alcohol use.

## Method

The Company-School Integration Center (*Centro de Integração Empresa-Escola* - CIEE) and the Federal University of São Paulo (*Unifesp*) developed an interactive website with free access on alcohol consumption among university students. The website can be accessed through the address www.ciee.org.br/portal/estudantes/inicial_pesq.asp

Participant recruitment (previously registered in the CIEE) was carried out by e-mail, and data collection was performed between October 2014 and March 2015. Only invited students had access to the survey. A total of 2,596 individuals accessed the website and responded to the survey. The inclusion criteria for this study were: being between 18 and 30 years old and being enrolled in any Institution of Higher Education (*Instituição de Ensino Superior* - IES). Participants who did not meet these criteria were able to complete the questionnaires, however their data were disregarded from the analyses (N=167). As a way of ensuring the veracity of participants’ responses, a question about the use of a fake drug was included^9^; university students who responded positively to this question had their data excluded from the analyzes (N=21). The final sample evaluated in this study corresponded to 2,408 university students (N=2,408).

### Instruments

The *Alcohol Use Disorders Identification Test* (AUDIT)^10^ was used to evaluate the alcohol consumption, which was previously validated among university students^11^ ^)^ and for the Brazilian population^12^, and adapted to refer to the last three months. On the other hand, eight questions were used on the consequences of alcohol consumption, based on the scale *Rutgers Alcohol Problem Index* (RAPI)^13^, with questions regarding the behavior of drinking and driving, getting a ride with someone who had consumed alcohol, nausea or vomiting, impulsive behavior, and a question about the participant’s assessment of how negative the consequences were. Regarding the problems associated with alcohol use, answers to questions 4 to 10 of the AUDIT were used.

### Ethical Considerations 

This study was submitted and approved (CEP: 429 170) by the Research Ethics Committee of the Federal University of São Paulo (CAAE: 22423513.4.0000.5505). A Free and Informed Consent Form was made available on the website’s homepage*.*

### Statistical analyses

The participants were classified into three groups according to the profile of alcohol consumption in the previous three months: no alcohol consumption; alcohol consumption without binging (NB), and alcohol consumption with binge drinking (BD).

Chi-square and one-way ANOVA statistical tests were used. Linear and logistic regression models were used to compare groups of alcohol use with and without binging. The primary outcomes evaluated were: total AUDIT score, total number of consequences associated with alcohol consumption, problems associated with alcohol consumption (AUDIT questions 4-8) and consequences of alcohol consumption. The secondary outcomes evaluated were: money spent on alcoholic beverages (R$), maximum number of drinks consumed per hour, and participant’s perception regarding how negative the consequences are for it. All regression models were adjusted according to gender, age, income, type of institution, age at first alcohol consumption, and age at the first episode of intoxication. A minimum level of 5% statistical significance was adopted. The analyses were performed using Stata software v.12.0.

## Results

More than half of the students were male (55.2%), residing in the South and Southeast Regions (54.2%), with monthly family income between 1 to 3 minimum wages (56.3%) and a mean age of 21.6 years (standard deviation - sd of 0.06). Approximately 85% of university students came from private institutions and had attended the university for 2.4 years on average (sd=0.02), while 54% of the students were from courses in the humanities area (Table 1). In addition, alcohol consumption frequency in the previous three months was 89.2%, and 51.6% of university students reported binge drinking. Among students who consumed alcohol, the majority consumed between 1 and 4 drinks (65.6%). We also found that the frequency of women was higher in the NB group, while males were more frequent in the BD group (x^2^(2)=39.13; p<0.001). Students from the Mid-west region reported less frequent use of alcohol, with or without patterns of binging (x^2^(4)=12.03; p=0.02), while students with a family income greater than 10 minimum wages were more prevalent in both the NB and BD groups (x^2^(8)=31.68; p<0.001). 

**Table 1:** Sociodemographic characteristics among individuals who did not consume alcohol, those who consumed without binging, and those who binged in the three months prior to the survey (N=2,408). São Paulo, SP, Brazil, 2014/2015.

		**Total (N=2,408)**	**Did not drink (N=261)**	**Did not binge (N=911)**	**Binged (N=1.236)**	**χ² or F**
**Gender - N (%)**						
	**Female**	**1,079 (44.8%)**	**125 (47.9%)**	**475 (52.1%)**	**479 (38.8%)**	39.13*
	**Male**	**1,329 (55.2%)**	**136 (52.1%)**	**436 (47.9%)**	**757 (61.3%)**	
**Age - mean (sd)** ^**†**^		**21,6 (0,06)**	**21.6 (0.18)**	**21.6 (0.10)**	**21.7 (0.08)**	**0.37**
**Region - N (%)**						
	**North and Northeast**	**651 (27.1%)**	**70 (26.8%)**	**212 (23.3%)**	**369 (29.9%)**	**12.03** ^**‡**^
	**Mid-west**	**226 (18.7%)**	**51 (19.5%)**	**174 (19.1%)**	**226 (18.3%)**	
	**South and Southeast**	**1,305 (54.2%)**	**140 (53.6%)**	**525 57.6%)**	**640 (51.8%)**	
**Income(R$) - N (%)**						
	**Not sure**	**138 (5.7%)**	**17 (6.5%)**	**33 (3.6%)**	**88 (7.1%)**	31.68*
	**1 to 3 minimum wages**	**1,355 (56.3%)**	**157 (60.2%)**	**557 (61.1%)**	**641 (51.9%)**	
	**3 to 5 minimum wages**	**435 (18.1%)**	**48 (18.4%)**	**153 (16.8%)**	**234 (18.9%)**	
	**5 to 10 minimum wages**	**337 (14.0%)**	**27 (10.3%)**	**126 (13.8%)**	**184 (14.9%)**	
	**10 or more minimum wages**	**143 (5.9%)**	**12 (4.6%)**	**42 (4.6%)**	**89 (7.2%)**	
**Institution - N (%)**						
	**Private**	**2,048 (85.1%)**	**227 (87.0%)**	**788 (86.5%)**	**1,033 (83.6%)**	**4.37**
	**Public**	**360 (15.0%)**	**34 (13.0%)**	**123 (13.5%)**	**203 (16.4%)**	
**Area of knowledge - N (%)**						
	**Biological sciences**	**157 (6.5%)**	**12 (4.6%)**	**57 (6.3%)**	**88 (7.1%)**	**3.75**
	**Exact sciences**	**952 (39.5%)**	**98 (37.6%)**	**358 (39.3%)**	**496 (40.1%)**	
	**Human sciences**	**1,299 (54.0%)**	**151 (57.9%)**	**496 (54.5%)**	**652 (52.8%)**	
**Year of the course - mean (sd)** ^**†**^		**2.4 (0,02)**	**2.4 (0.07)**	**2.4 (0.03)**	**2.4 (0.03)**	**1.64**

*p<0.001; †sd: standard deviation; ‡p<0.01; minimum wages in 2014, Brazil.

The majority of university students were classified as Low Risk by the AUDIT (77.5%), and the frequency of risky use was significantly higher in the BD group (31.3% - x^2^(3)=344.64; p<0.001). On the other hand, students of the BD group reported consuming a significantly higher maximum number of drinks (F(1,2145)=518.71; p*<*0.001) and spending more on alcoholic beverages (F(1,2145)=109.71; p*<*0.001) than the NB group (Table 2).

**Table 2:** Characteristics of the pattern of alcohol use among individuals who did not consume alcohol, those who consumed without binging and those who binged in the three months prior to the survey (N=2,408). São Paulo, SP, Brazil, 2014/2015.

		**Total (N=2.147)**	**Did not binge (N=911)**	**Binged (N=1.236)**	**χ² or F**
Age at first alcohol consumption - mean (sd)*		**16.1 (0.05)**	**16.6 (0.08)**	**15.7 (0.06)**	**66.8** ^**†**^
Age at the first episode of intoxication - mean (sd)*		**17.7 (0.05)**	**18.2 (0.08)**	**17.3 (0.07)**	**79.1** ^**†**^
AUDIT score - mean (sd)*		**5.1 (0.09)**	**2.2 (0.06)**	**7.3 (0.12)**	**1079.4** ^**†**^
**AUDIT Risk Zone - N (%)**					
	**Low Risk**	**1.663 (77.5%)**	**883 (96.9%)**	**780 (63.1%)**	**344,6** ^**†**^
	**Hazardous drinking**	**415 (19.3%)**	**28 (3.1%)**	**387 (31.3%)**	
	**Harmful drinking**	**41 (1.9%)**	**0 (0)**	**41 (3.3%)**	
	**Dependence**	**28 (1.3%)**	**0 (0)**	**28 (2.3%)**	
Maximum number of drinks consumed - mean (sd)*		**6.5 (0.13)**	**3.4 (0.11)**	**8.9 (0.19)**	**518.7** ^**†**^
Maximum number of drinks consumed per hour - mean (sd)*		**1.8 (0.03)**	**1.3 (0.04)**	**2.1 (0.05)**	**123.2** ^**†**^
Money spent on alcoholic beverages - mean (sd)*		**54.0 (1.62)**	**34.7 (1.60)**	**68.2 (2.47)**	**109.7** ^**†**^

*sd: standard deviation; †p<0.001.

The consequences and problems associated with alcohol consumption in the previous three months are presented in Table 3. All consequences and problems were more prevalent in the BD group. However, the same group evaluated the consequences as being less negative on average (F(1,2145)=6.40, p<0.01). Table 4 presents the logistic regression models comparing the participants from NB and BD groups, predicting the consequences and problems associated with alcohol use. For all evaluated outcomes, the models showed that university students in the BD group were more likely to report some of the consequences or problems associated with alcohol use.

**Table 3:** Prevalence of consequences and problems associated with alcohol use among individuals who did not consume alcohol, those who consumed without binging and those who binged in the three months prior to the survey (N=2,408). São Paulo, SP, Brazil, 2014/2015.

		**Total (N=2.147) Yes**	**Did not binge (N=911) Yes**	**Binged (N=1.236) Yes**	**χ² or F**
**Consequences of consuming alcohol - N (%)**					
	**Drove under the influence of alcohol**	**398 (18.5)**	**82 (9.0)**	**316 (25.6)**	95.3*
	**Got a ride with someone who drank**	**1.245 (58.0)**	**444 (48.7)**	**801 (64.8)**	55.6*
	**Academic problems**	**232 (10.8)**	**40 (4.4)**	**192 (15.5)**	67.6*
	**Problems with their boyfriend/girlfriend or a close relative**	**402 (18.7)**	**114 (12.5)**	**288 (23.3)**	40.1*
	**Nausea or vomiting**	**979 (45.6)**	**286 (31.4)**	**693 (56.1)**	128.7*
	**Physical fights**	**123 (5.7)**	**25 (2.7)**	**98 (7.9)**	26.1*
	**Sexual intercourse without using a condom**	**444 (20.7)**	**127 (13.9)**	**317 (25.7)**	43.8*
	**Acted impulsively and regretted it**	**638 (29.7)**	**153 (16.8)**	**485 (39.2)**	126.5*
**Number of consequences associated with alcohol consumption - mean (sd)** ^**†**^		**2.1 (0,04)**	**1.4 (0.05)**	**2.6 (0.05)**	243.0*
**How negative these consequences are for the participant - mean (sd)** ^**†**^		**3.7 (0,08)**	**3.9 (0.13)**	**3.5 (0.09)**	**6.4** ^**‡**^
**Problems associated with alcohol use - N (%)**					
	**Thought you were not able to stop drinking**	**306 (14.3)**	**46 (5.1)**	**260 (21.0)**	109.7*
	**Could not do what was expected**	**309 (14.4)**	**44 (4.8)**	**265 (21.4)**	117.4*
	**Needed to drink/have a drink in the morning to feel good**	**94 (4.4)**	**13 (1.4)**	**81 (6.6)**	32.9*
	**Felt guilty or remorse after drinking**	**563 (26.2)**	**141 (15.5)**	**422 (34.1)**	94.4*
	**Was unable to remember what happened/blacked out**	**463 (21.6)**	**67 (7.4)**	**396 (32.0)**	188.9*
	**Have you caused injury or damage to someone/something**	**387 (18.0)**	**8 8 (9.7)**	**299 (24.2)**	74.9*
	**Someone has been concerned with your consumption**	**344 (16.0)**	**66 (7.2)**	**278 (22.5)**	90.6*

*p<0.001; †sd: standard deviation; ‡p<0.01.

**Table 4:** Adjusted logistic regression models* comparing university students who consumed alcohol without binging (reference) and those who binged in the previous three months (N=2,146). São Paulo, SP, Brazil, 2015.

		**Binge drinking (N=1.236)**	
		**OR** ^**†**^ **( 95% CI)** ^**‡**^	**aOR** ^**§**^ **(95% CI)** ^**‡**^
**Consequences of alcohol consumption**			
	**Drove under the influence of alcohol**	**3.5 (2.7-4.5)** ^**||**^	**2.6 (2.0-3.5)** ^**||**^
	**Got a ride with someone who drank**	**1.9 (1.6-2.3)** ^**||**^	**1.8 (1.5-2.2)** ^**||**^
	**Academic problems**	**4.0 (2.8-5.7)** ^**||**^	**3.4 (2.4-4.9)** ^**||**^
	**Problems with their boyfriend/girlfriend or a close relative**	**2.1 (1.7-2.7)** ^**||**^	**1.9 (1.4-2.4)** ^**||**^
	**Nausea or vomiting**	**2.8 (2.3-3.3)** ^**||**^	**2.8 (2.3-3.3)** ^**||**^
	**Physical fights**	**3.1 (2.0-4.8)** ^**||**^	**2.6 (1.6-4.1)** ^**||**^
	**Sexual intercourse without using a condom**	**2.1 (1.7-2.7)** ^**||**^	**1.8 (1.5-2.3)** ^**||**^
	**Acted impulsively and regretted it**	**3.2 (2.6-3.9)** ^**||**^	**2.9 (2.3-3.6)** ^**||**^
**Problems associated with alcohol use**			
	**Thought they were not able to stop drinking**	**5.0 (3.6-6.9)** ^**||**^	**4.6 (3.3-6.5)** ^**||**^
	**Could not do what was expected**	**5.4 (3.9-7.5)** ^**||**^	**4.7 (3.4-6.7)** ^**||**^
	**Needed to drink/have a drink in the morning to feel good**	**4.8 (2.7-8.8)** ^**||**^	**5.0 (2.7-9.1)** ^**||**^
	**Felt guilty or remorse after drinking**	**2.8 (2.3-3.5)** ^**||**^	**2.6 (2.1-3.2)** ^**||**^
	**Was unable to remember what happened/blacked out**	**5.9 (4.5-7.8)** ^**||**^	**5.4 (4.1-7.2)** ^**||**^
	**Has caused injury or damage to someone or something**	**3.0 (2.3-3.9)** ^**||**^	**2.4 (1.8-3.2)** ^**||**^
	**Has worried about their own consumption habit**	**3.7 (2.8-4.9)** ^**||**^	**3.0 (2.2-4.1)** ^**||**^

*Adjusted for gender, age, income, institution, age at first alcohol consumption and age at first intoxication; †OR: *Odds Ratio*; ‡95% CI: 95% Confidence interval; §aOR: adjusted *Odds Ratio*; || p<0.001.

The linear regression models presented in Table 5 indicate that compared to the NB group, university students had an average score of 4.60 on the AUDIT; one more negative consequence; a maximum drink consumption around 0.65 more drinks per hour and an spent an average of R$29.68 more on beverages. Although the BD group presented more negative consequences, this group showed a negative perception of the consequences of 0.45 points less on average than the NB group.

**Table 5:** Adjusted linear regression models^*^ comparing university students who consumed alcohol without binging (reference) and those who binged in the previous three months (N=2,146). São Paulo, SP, Brazil, 2015.

	**b**	**Standard error b**	**β**	**p value**	**95% CI** ^**†**^ **inferior**	**95% CI** ^**†**^ **superior**	**R** ^**2**^
**AUDIT Score**	**4.60**	**0.16**	**0.53**	**0.000**	**4.29**	**4.90**	**0.38**
**Number of consequences associated with alcohol consumption**	**1.01**	**0.08**	**0.27**	**0.000**	**0.85**	**1.16**	**0.15**
**How negative these consequences are for the participant**	**-0.45**	**0.16**	**-0.06**	**0.005**	**-0.75**	**-0.14**	**0.01**
**Maximum number of doses/drinks consumed per hour**	**0.65**	**0.07**	**0.20**	**0.000**	**0.51**	**0.79**	**0.08**
**Spending on alcoholic beverages (R$)**	**29.68**	**3.30**	**0.20**	**0.000**	**23.21**	**36.16**	**0.07**

*Adjusted for gender, age, income, institution, age at first alcohol consumption, and age at first intoxication; †95% CI: 95% Confidence interval.

## Discussion 

The present study indicated that in the sample of Brazilian university students who accessed a website about alcohol use, individuals who reported binge drinking in the previous three months had experienced more negative consequences associated to drinking, as well as a greater incidence of problems associated to it and greater financial expenditure on alcoholic beverages. Nonetheless, these students perceived the consequences reported by them as not so negative. The results corroborate the three initially proposed hypotheses: university students who consume alcohol and binge would present higher scores on the AUDIT, a greater number of negative consequences associated with alcohol use and would be more likely to report problems or consequences associated with drinking. 

In relation to alcohol use in the previous three months, a frequency of 89.2% was observed in this study, which is higher than use in life (86.2%) reported in the main national reference on the topic: I National Survey on the Use of Alcohol, Tobacco and Other Drugs among University Students of the 27 Brazilian Capitals. The frequency of the binge drinking in the previous three months in this study (51.6%) was also higher than that observed use during the last year in the Survey (43.7%)^3^. Data from North American studies indicate the prevalence of binging within the last two weeks (35%), showed no major changes since the early 1990s^14^. On the other hand, the direct comparison between the data of this work with those of other studies has limitations, since different methods of data collection were used, in addition to referring to frequency data with different temporal cut-outs. In addition, data collection in this study was carried out via the internet, while in the Survey it was carried out from the collective application of a printed questionnaire during class time. The use of the Internet for this type of procedure can attenuate the subjects’ embarrassment regarding the use of drugs^15^, possibly reflecting in a higher frequency of alcohol use. Another relevant aspect: during recruitment, participants were invited to access a survey to learn more about their current alcohol consumption habits. Thus, as previously mentioned, it is possible that this sample presents an underrepresentation of university students who do not consume alcoholic beverages. 

The frequency of men was higher in the BD group, while women were more prevalent in the NB group. These data suggest that women do not seem to abstain from alcohol use, however, they binge drink less frequently than men. This data corroborates some findings that binge drinking among men is greater than among women^14^ ^,^ ^16^. The same data is similar to those of international studies indicating a higher prevalence of binging among men^17^. In this study, the North-Northeast Regions showed a higher frequency of alcohol use in the binge pattern, given the similarity observed in the National Survey^3^. The variable of family income of 10 or more minimum wages was associated with alcohol consumption with and without binging. An earlier study conducted among private high school students in São Paulo found a higher frequency of alcohol use, including binge drinking among higher socioeconomic classes^18^. 

The behavior frequency of getting a ride with someone who has consumed alcoholic beverages was approximately three times greater than drinking and driving behavior. *Lei Seca* (Dry Law) in Brazil was established in 2008 and updated in 2012, setting penalties for people driving under the influence of alcohol. Despite the 45% reduction in the frequency of adults driving after binging practices between 2007 and 2013^19^, 60.2% of the drivers had been passengers of drivers who also had consumed alcoholic beverages^20^. Among students 11 to 21 years of age, 8% reported drinking and driving, while 32% reported getting a ride in the last 12 months with someone who had consumed alcoholic beverages, and 16.6% assumed they had taken a ride with a driver who had had too much to drink to drive safely^21^. These data suggest that despite the reduction in drinking and driving behavior, the frequency of individuals who are at risk of traffic accidents is still high, considering that even when they do not drink and drive themselves, many get rides with someone who has consumed alcoholic beverages.

Binge drinking was associated with a greater chance of reporting any of the consequences or problems assessed in this study. On the occasions that they consumed the most alcohol, university students in the BD group had an average intake of 2.1 drinks/hour, for an average of 5.2 hours. Such data suggest that these university students presented relevant signs of drunkenness. In this sense, it is reasonable to think that the respective group is more likely to engage in risky behaviors. Binge drinking is associated with short- and long-term cognitive, physiological, social, and academic problems. For example, binge drinking by college students has been associated with a greater chance of experiencing more problems during the university period, or alcohol abuse and dependence after 10 years^22^. In addition, this pattern is associated with numerous other acute negative consequences^5^. 

Although the college students in the BD group had more consequences associated with alcohol use, they evaluated the consequences as less negative. It would be expected that the greater the number of consequences experienced, the greater the perception of their negative impact. However, although few studies have explored perceptions about the consequences of alcohol consumption, a lower negative evaluation of behavioral/physical consequences by university students, has previously been associated with a greater number of problems related to alcohol consumption^23^. Another study also indicated that university students who consume alcohol in excess tend to perceive some consequences (hangovers, blackouts, vomiting, missing class or work), as more positive than negative experiences^24^. Thus, consequences traditionally perceived as negative by researchers may assume a positive character among some students, which can act in a way to reinforce the behavior of alcohol use ^(^ ^25^.

Among limitations of this study, we can point out that because it is a cross-sectional study, its results do not allow inferring a causal relationship. Although the results came from a large national sample with distribution among public and private institutions and with very similar to data from the Census of Higher Education^1^, the results of this study are not representative of the Brazilian university student population, since it was not designed to obtain representative sampling from this population. Another limitation is the low response rate of students from the Southern Region, a characteristic of CIEE’s own e-mail database due to the decentralization of some of the company’s regional ones. Due to the characteristics of the invitation to access the website, there is also possibly an underrepresentation of university students who do not consume alcoholic beverages.

## Conclusion

Among the study participants, we observed reports of binging alcohol consumption in a considerable portion of the sample. Compared to alcohol consumption without binging, the variable binge drinking was significantly associated with a greater chance of reporting several problems and negative consequences related to alcohol consumption. These results suggest a specific risk group for problems associated with alcohol use. In order to reduce the impact of the problems associated with this consumption, the information in this study should be considered in future public or institutional policies focused on Brazilian university students. 
